# The shared genetic architecture between epidemiological and behavioral traits with lung cancer

**DOI:** 10.1038/s41598-021-96685-x

**Published:** 2021-09-02

**Authors:** Rowland W. Pettit, Jinyoung Byun, Younghun Han, Quinn T. Ostrom, Jacob Edelson, Kyle M. Walsh, Melissa L. Bondy, Rayjean J. Hung, James D. McKay, Christopher I. Amos

**Affiliations:** 1 Institute for Clinical and Translational Research, Baylor College of Medicine, One Baylor Plaza, Houston, TX 77030 USA; 2 Section of Epidemiology and Population Sciences, Department of Medicine, Baylor College of Medicine, Houston, TX USA; 3 Duke Cancer Institute, Duke University Medical Center, Durham, NC USA; 4 Department of Biomedical Data Science, School of Medicine, Stanford University, Stanford, CA USA; 5 Department of Epidemiology and Population Health, School of Medicine, Stanford University, Stanford, CA USA; 6 Lunenfeld-Tanenbaum Research Institute, Sinai Health System, Toronto, Canada; 7 Division of Epidemiology, Dalla Lana School of Public Health, University of Toronto, Toronto, Canada; 8 Section of Genetics, International Agency for Research on Cancer, World Health Organization, Lyon, France; 9 Dan L Duncan Comprehensive Cancer Center, Baylor College of Medicine, Houston, TX USA

**Keywords:** Bioinformatics, Genome-wide association studies, Cancer genomics, Lung cancer, Non-small-cell lung cancer, Small-cell lung cancer

## Abstract

The complex polygenic nature of lung cancer is not fully characterized. Our study seeks to identify novel phenotypes associated with lung cancer using cross-trait linkage disequilibrium score regression (LDSR). We measured pairwise genetic correlation (r_g_) and SNP heritability (h^2^) between 347 traits and lung cancer risk using genome-wide association study summary statistics from the UKBB and OncoArray consortium. Further, we conducted analysis after removing genomic regions previously associated with smoking behaviors to mitigate potential confounding effects. We found significant negative genetic correlations between lung cancer risk and dietary behaviors, fitness metrics, educational attainment, and other psychosocial traits. Alcohol taken with meals (r_g_ = − 0.41, h^2^ = 0.10, p = 1.33 × 10^–16^), increased fluid intelligence scores (r_g_ = − 0.25, h^2^ = 0.22, p = 4.54 × 10^–8^), and the age at which full time education was completed (r_g_ = − 0.45, h^2^ = 0.11, p = 1.24 × 10^–20^) demonstrated negative genetic correlation with lung cancer susceptibility. The body mass index was positively correlated with lung cancer risk (r_g_ = 0.20, h^2^ = 0.25, p = 2.61 × 10^–9^). This analysis reveals shared genetic architecture between several traits and lung cancer predisposition. Future work should test for causal relationships and investigate common underlying genetic mechanisms across these genetically correlated traits.

## Introduction

In 2020 ~ 230,000 lung cancer cases will be diagnosed in the US, and ~ 140,000 people will die from their disease^1^. In total, this morbidity ranks lung cancer as the leading cause of cancer-related deaths in the United States. Our current understanding of lung cancer is that it is a multi-factorial disease in which tumorigenesis results from inherited genetic variants^2,3^, sustained environmental exposures^4^, and stochastic somatic mutations^5^. Environmental exposures associated with an increased risk of developing lung cancer are numerous and include cigarette smoke^6^, radon^7^, individual diet^8^, pollution in the atmosphere^9^, metallurgy^10^, and indoor pollution from cooking or heating with solid fuels^11^. The most significant contributor to lung cancer development is due to tobacco smoking^12^. However, clustering of lung cancer cases in families beyond a level that could be explained by shared environmental exposures to tobacco smoke or pollution supports a role of genetic factors contributing to disease risk^13–17^. Investigations into the precise tumorigenic mechanisms behind the familial aggregation of lung cancer are complicated by genetic polygenicity^18,19^, whereby a combination of multiple genes contributes to risk.

Genome-wide association studies (GWAS), which examine millions of single nucleotide polymorphisms (SNPs) for association with a trait of interest, are helpful for deciphering the genetic architecture of complex diseases^2^. GWAS is not without limitations, and behavioral traits that are genetically influenced can mediate observed associations between SNPs and lung cancer risk^20–23^. GWAS analysis can be further confounded when unknown population stratification or cryptic relatedness exists in the underlying data^24^. Prior GWAS investigations in lung cancer have revealed unique loci with strong statistical significance, yet, these regional associations vary across histological subtypes of lung cancer^2^. On top of heterogeneity between histological subtypes, known lung cancer risk loci only account for a minor proportion of the total estimated heritability of lung cancer, indicating a substantial proportion of the heritable causes^25^ of lung cancer remains unidentified.

A more comprehensive approach to understanding tumorigenic mechanisms may be fruitful. Focused work into understanding the genetic architecture behind disease co-development may be more informative than studying individual phenotypes^26,27^. A knowledge gap exists today to quantify the extent that other diseases, environmental exposures, and phenotypic traits correlate with a predisposition to lung cancer. A novel regression statistical framework, known as cross-trait linkage disequilibrium score regression (LDSR), may be employed to fill this gap in knowledge. LDSR uses GWAS summary statistics to identify genome-wide genetic correlations between phenotypes of interest^28^. The similarity of measured SNP effect estimates reported by GWAS summary statistics are compared between traits. LDSR allows for accurate calculations of genetic co-correlation (r_g_) between phenotypes while minimizing effects from selection biases in the recruitment of comparable controls from the same source population^24^. Use of this method can identify correlations in the genetic architecture between traits, allowing etiological insights to be gleaned.

Here, we quantify the association between genetically influenced epidemiological and behavioral traits and the risk of lung cancer. We use summary statistics generated by prior lung cancer GWAS and use LDSR to estimate cross-trait genetic correlations with lung cancer. We additionally evaluate how these traits correlate with each of the major histological subtypes of lung cancer—adenocarcinoma, squamous cell carcinoma, and small cell carcinoma, and further evaluate associations in ever- and never-smokers. We aimed to confirm prior associations with lung cancer and to identify novel phenotypic associations from GWAS datasets.

## Methods

### Summary statistics for lung cancer

This work is a continuation of efforts conducted by the Transdisciplinary Research of Cancer in Lung of the International Lung Cancer Consortium (TRICL-ILCCO)^29^ and the OncoArray Consortium^30^. The TRICL-OncoArray Consortium has previously published GWAS summary statistics results after a meta-analysis of lung cancer GWAS. The complete methods have been published previously^29,30^, but are presented here in brief. Lung cancer patients and healthy controls with no personal lung cancer history were recruited after individual institutional IRB approval and informed consent for genotyping. Genotyping occurred using the Illumina OncoArray-500K BeadChip of 533,631 SNPs. Standard quality control measures were implemented to exclude underperforming samples and SNPs^29^. Individuals and SNPs with genotyping call-rates < 95% were removed. Genotype imputation was conducted using the reference dataset of the 1000 Genomes Project Phase 3 (October 2014). The more common variant was included during the imputation process for positions with > 2 alleles. After imputation and quality control processes, 502,933 SNPs from 29,266 lung cancer patients and 56,450 healthy controls of European ancestry were incorporated into a meta-analysis^29^. Amongst the lung cancer cases, 11,273 cases of adenocarcinoma, 2,664 cases of small cell carcinoma, and 7,426 cases of squamous cell carcinoma were represented as histological subtypes (Supplementary Table 1). We obtained and utilized the summary statistics from the TRICL-ILCCO GWAS meta-analysis^29^ regarding lung cancer, the histological subtypes of lung cancer, and summary statistics for 'ever' vs. 'never '-smoking status sub-cohorts.

### Phenotype and exposure accession with United Kingdom Biobank genome-wide association studies

GWAS summary statistics for cross-trait LDSR analyses were obtained from the United Kingdom Biobank (UKBB). The UKBB is a national and international health repository^31^. Since its inception in 2006, the UKBB has collected clinical and genotypic data for 500,000 adult participants across 22 sites in the United Kingdom^31^. Participants in this longitudinal project were age 40–69 at enrollment. Initial relevant information is gathered by clinical exam, questionnaire, and biospecimen sampling. Participants will be followed for 30+ years. Periodically, follow-up health data are obtained by a linked unique encrypted identifier with electronic health records from the UK National Health Service (NHS). Each of the > 500,000 participants in the UKBB has been genotyped, 90% of which were genotyped using a custom Affymetrix UKBB Axiom array. This array assayed ~ 850,000 variants across the genome, which were used to impute 9.1 million SNPs with satisfactory quality control measures in place. These imputation procedures are conducted by the Wellcome Trust Center for Human Genetics and are conducted internally at the UKBB before the data release. GWAS was conducted from these imputed data, and summary level statistics were made publicly available (https://nealelab.github.io/UKBB_ldsc/downloads.html#reference_files). We obtained all of our GWAS summary statistics from the second batch of UKBB GWAS results published online and updated in August 2018.

### Harmonization and quality control with SNP filtering

We harmonized the obtained publicly available GWAS summary statistics. Our final dataset included summary statistics for selected epidemiological and individual lifestyle traits, including alcohol use and fitness activity levels and routines. The final dataset also included biometric measurements, including BMI and body fat percentage measurements. Reported educational attainment, employment status, workplace environment, and psychological experiences were also included. These obtained UKBB summary statistics contained SNP-level effect sizes (beta) for each trait, with Z-scores calculated by dividing SNP effect sizes by their standard error. To harmonize these datasets, and as an additional quality control measure, we filtered the imputed SNPs from the UKBB to include only those autosomal SNPs with a minor allele frequency greater than 0.01 and imputation quality INFO score greater than 0.90. We further removed SNPs from our harmonized data set that were not in HapMap3 with a minor allele frequency less than 5% in European populations, in line with previously published methods^24,32^.

### Estimating pairwise genetic correlations and heritability

With this information, we estimated genome-wide SNP heritability using LDSR. Additionally, we used LDSR to compute the pairwise genetic correlation between each of the UKBB traits with lung risk from the TRICL-OncoArray consortium. LDSR calculates genetic correlation by regressing the product of SNP z scores (Z_UKBB_ * Z_TRICL-OncoArray_) against the SNP's calculated linkage disequilibrium score^24^. The slope of this regression accurately estimates the genetic covariance between two traits. Genetic covariance is converted to a genetic correlation between traits by normalizing genetic covariance by the calculated heritability of each of the two compared traits. The heritability of a trait can be thought of as the genetic covariance of a trait with itself and ultimately represents the proportion of a trait that genetic effects can explain^28^.

LDSR mitigates potential biases from population stratification^19^ and cryptic relatedness^24^ by modeling an intercept term that accounts for any genomic inflation. We applied a cross-trait LDSR model that included an intercept in these analyses to account for hidden biases that may exist between reference and target populations, especially those that may arise due to the instability of linkage disequilibrium scores in European populations and sub-populations^24,33^.

We used LDSR to calculate the genetic correlations between lung cancer risk and traits of interest. We additionally performed LDSR for each of the histological subtypes of lung cancer, including small cell carcinoma, squamous cell carcinoma, and adenocarcinoma. Further, we performed LDSR between traits of interest and lung cancer risk in ever- and never-smoker subgroups. Individuals who reported having smoked fewer than 100 cigarettes throughout their lives were defined as "never smokers," and those who had smoked more than 100 cigarettes in their life as "ever smokers"^29^. We stratified both lung cancer cases and controls by smoking status for these analyses.

### Removal of known regions related to smoking behaviors

If a trait shows a genetic correlation with lung cancer in LDSR analyses, this does not necessarily imply a causal relationship. Indeed, both the trait and lung cancer risk may be jointly influenced by a third, unmodeled trait that independently influences each. Notably, smoking status has the potential to confound our associations (e.g., the genetic correlation between lung cancer risk and emphysema risk would likely be attributable to the effect of smoking on both diseases)^34–38^. In addition to stratifying our LDSR analyses by 'never' and 'ever' smoking status as available from the TRICL-OncoArray Consortium, we also excluded genomic loci previously associated with smoking behaviors. A recent meta-analysis quantified the effect of SNPs on several smoking behaviors, including "age of initiation of smoking", "cigarettes per day", "smoking cessation", and "smoking initiation"^39^. These authors used a conditional analysis method^40^ to identify SNPs independently associated with at least one of these smoking related traits. Applying a predetermined genome-wide significance threshold of p < 5 × 10^–8^, 467 SNPs were found to be associated with smoking related traits^39^. We repeated our LDSR analyses after removing each of these 467 smoking-related SNPs from our summary statistics. Specifically, we identified the sentinel variant from the meta-analysis and removed all SNPs within ± 500 kb. SNPs that were filtered at this step appear in Supplementary Table 2, which also annotates the upper and lower bounds of the genomic regions removed. Changes in the number of SNPs included and excluded from this analysis, per histological subgroup and lifetime smoking status, appear in Supplementary Table 3. Quantile–Quantile plots of the p-values observed from the TRICL-OncoArray meta-analysis before and after removing smoking-related SNPs may be appreciated in Supplementary Figure 1.

**Figure 1:**
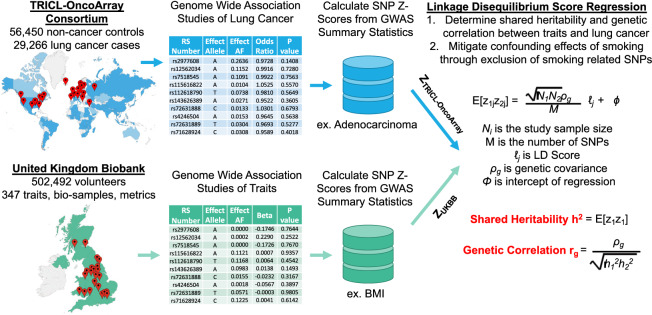
Graphical representation of the analytical workflow, including datasets utilized and analyses performed. Maps rendered with Tableau Desktop, 2021 Tableau Software, LLC, https://www.tableau.com/.

We summarized and presented these methods graphically in Fig. 1. Multiple comparisons are conducted in executing these methods. We tested 347 traits and associated them to determine their genetic predispositions to develop overall lung cancers, adenocarcinomas, squamous cell carcinomas, and small cell carcinomas. Additionally, we tested these traits for associations in 'never' or 'ever' smoking populations. These 2082 independent tests were conducted twice, before and after the removal of smoking-related SNPs. In total, 4164 comparisons were performed. Using a stringent Bonferroni correction, we set our adjusted P value significance cutoff threshold to be less than 1.2 × 10^–5^, or − log_10_(P) > 4.92. Here we report the trait associations with significance metrics less than the Bonferroni adjustment. In Supplementary Table 5, we present the heritability, genetic correlations, significance values for each comparison conducted. In this table, we further provide LDSR confidence thresholds and heritability thresholds for each UKBB trait. We finally offer a direct uniform resource locator link for each UKBB trait, allowing for ease of inquiry into trait type counts, inclusion criteria, distribution histograms, and other relevant metrics.

## Results

### Heritability of lung cancer and its histological subtypes

Overall, we found the heritability of lung cancer to be 8.3% ± a standard error of 1.3%, which persisted even after smoking-related regions were removed (6.9 ± 0.8%). Stratifying by 'ever' and 'never' smoking status, we estimate the overall lung cancer heritability to be 10.0 ± 2.1% in 'ever' smokers and 3.0 ± 4.8% in 'never' smokers. After removing smoking-related SNPs, the estimated heritability in 'ever' smokers was 7.7 ± 1.4%, and in 'never' smokers was 2.9 ± 4.7%. Stratifying amongst the histological subtypes of lung cancer, and including all SNPs, adenocarcinoma heritability was 6.7 ± 1.0%, small cell lung cancer heritability was 10.5 ± 1.9%, and squamous cell carcinoma of the lung had an estimated heritability of 5.2 ± 1.1%. After removing smoking-related SNPs, heritability estimates fell to 6.2 ± 0.9% (adenocarcinoma), 9.4 ± 2.0% (small cell), and 4.4 ± 0.9% (squamous cell carcinoma of the lung) (Supplementary Table 4). The heritability of each of 347 traits modeled using LDSR appears in Supplementary Table 5.

### Heritability and genetic correlations between lung cancer and alcohol use

**Figure 2:**
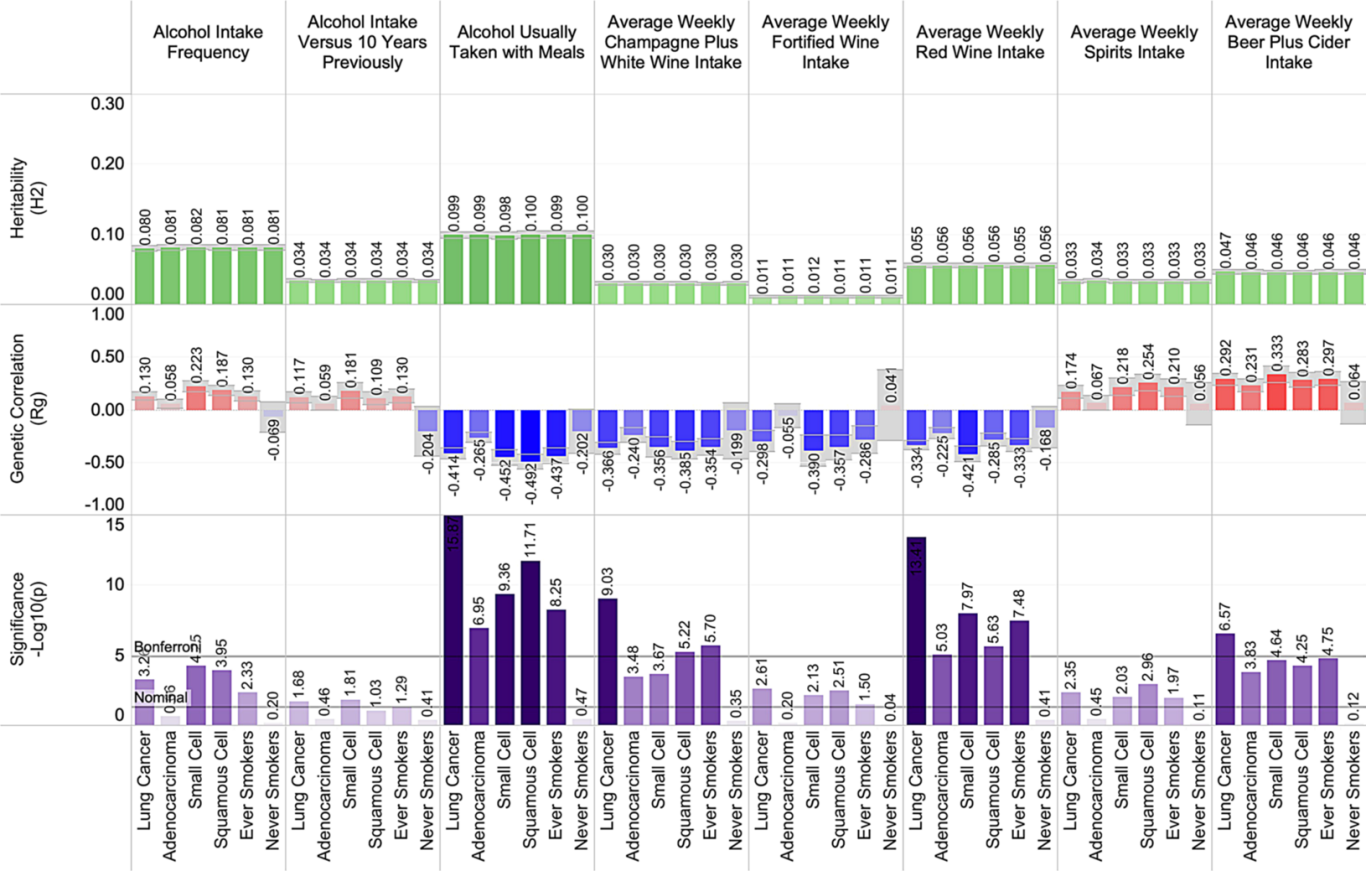
The shared heritability and genetic correlation between alcohol use and lung cancer.

Using cross-trait LDSR, we found that "alcohol usually taken with meals" had an estimated heritability of 0.1 and demonstrated a negative genetic correlation with lung cancer risk across histological subtypes and smoking status. Specifically, "alcohol usually taken with meals" demonstrated a − 0.41 genetic correlation (r_g_) with all lung cancer (p = 1.33 × 10^–16^). These findings remained consistent after excluding regions associated with smoking behaviors (r_g †_ = − 0.37, p_†_ = 4.46 × 10^–13^). Further investigation revealed that average weekly beer plus cider intake demonstrated positive genetic correlation with lung cancer susceptibility (pre-removal: r_g_ = 0.29, p = 2.68 × 10^–7^; post-removal: r_g †_ = 0.29, p_†_ = 9.87 × 10^–7^), whereas average weekly red wine intake demonstrated negative genetic correlation with overall lung cancer susceptibility (r_g_ = − 0.33, p = 3.90 × 10^–14^; r_g †_ = − 0.31, p_†_ = 3.08 × 10^–9^). These findings were consistent across histological subtypes (Supplementary Table 5). A summary of the significant alcohol-related associations is presented in Fig. 2, and the results from association testing for all traits are included in Supplementary Table 5. All results from our LDSR analyses are publicly hosted and available for interactive viewing at https://public.tableau.com/profile/rowland.pettit.

### Heritability and genetic correlations between lung cancer and education/employment

**Figure 3:**
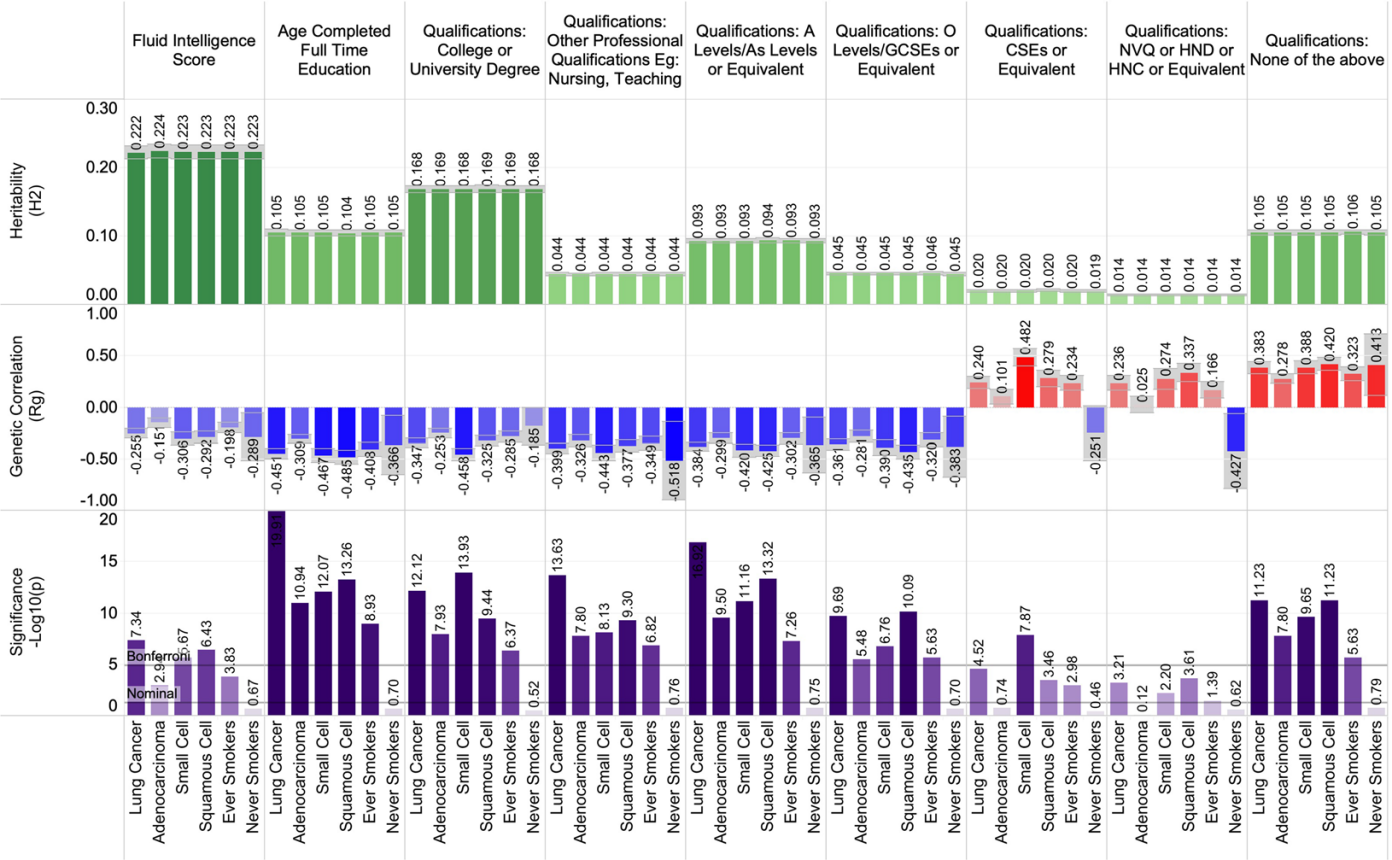
The shared heritability and genetic correlation between education and employment with lung cancer.

Education and employment statuses were genetically correlated with lung cancer susceptibility. As this self-reported personal characteristic information comes from the UK biobank, educational ascertainment metrics follow the United Kingdom advanced learning schemas. These analyses found that total years of education, obtaining a college or university degree, earning other advanced professional qualifications such as nursing or teaching roles, gaining “A” level qualification, or earning general certificates of secondary education all demonstrated significant negative genetic correlation with lung cancer susceptibility (Fig. 3). These trends persisted across histological subtypes, but associations were not statistically significant among ‘never’ smokers. Here we highlight reported correlations for “age completed full time education” with overall lung cancer, before and after removal of smoking-associated genomic regions (r_g_ = − 0.45, p = 1.24 × 10^–20^; r_g †_ = − 0.43, p_†_ = 1.06 × 10^–19^), small cell lung cancer (r_g_ = − 0.47, p = 8.55 × 10^–13^; r_g †_ = − 0.45, p_†_ = 6.20 × 10^–9^), squamous cell lung cancer (r_g_ = − 0.49, p = 5.46 × 10^–14^; r_g †_ = − 0.46, p_†_ = 8.40 × 10^–10^), adenocarcinoma (r_g_ = − 0.31, p = 1.15 × 10^–11^; r_g †_ = − 0.27, p_†_ = 3.04 × 10^–7^), ‘ever’ smokers (r_g_ = − 0.41, p = 1.17 × 10^–9^; r_g †_ = − 0.41, p_†_ = 4.51 × 10^–10^), and ‘never’ smokers (r_g_ = − 0.37, p = 0.20; r_g †_ = − 0.33, p_†_ = 0.11).

In contrast, obtaining none of the previously mentioned academic qualifications demonstrated a positive genetic correlation with lung cancer susceptibility, which was strongest in overall lung cancer (r_g_ = 0.38, p = 5.91 × 10^–12^; r_g †_ = 0.38, p_†_ = 3.78 × 10^–16^), and the trend held across histological subtypes and in 'ever' smokers. Fluid intelligence scores were genetically correlated with decreased lung cancer susceptibility across all histological and smoking status sub-classifications (overall r_g_ = − 0.25, p = 4.54 × 10^–8^) but did not reach statistical significance in 'never' smokers. The calculated Townsend deprivation index^41^, which is a metric combining the census demographics of car ownership, household overcrowding, household employment status, and house ownership, demonstrated significant increased genetic predisposition with lung cancers (overall lung cancer r_g_ = ﻿0.35, p = 1.03 × 10^–10^; r_g †_ =  ﻿0.28, p_†_ = 9.61 × 10^–6^). A summary of the significant education and employment-related associations is presented in Fig. 3.

### Heritability and genetic correlations between lung cancer and fitness metrics

**Figure 4:**
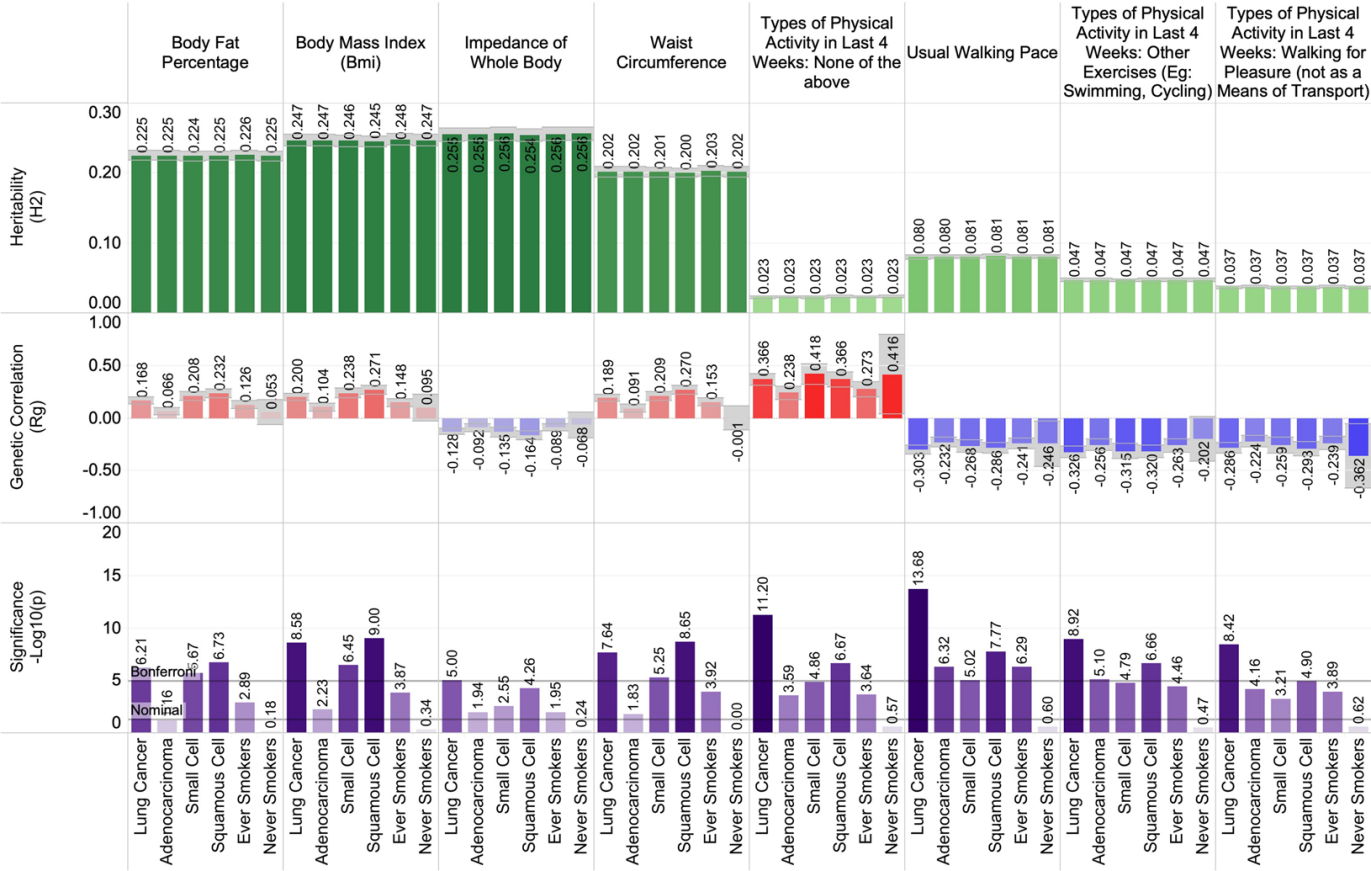
The shared heritability and genetic correlation between fitness with lung cancer.

Measured and reported fitness metrics were genetically correlated with lung cancer susceptibility. Increased body fat percentage, impedance of the whole body, waist circumference, and increased body mass index (BMI) correlated positively with lung cancer susceptibility. Highlighting BMI, positive genetic correlations were observed for overall lung cancer (r_g_ = 0.20, p = 2.61 × 10^–9^; r_g †_ = 0.19, p_†_ = 3.23 × 10^–8^) as well as across small cell lung carcinoma (r_g_ = 0.24, p = 3.54 × 10^–7^; r_g †_ = 0.24, p_†_ = 5.27 × 10^–5^), and squamous cell carcinoma (r_g_ = 0.27, p = 9.91 × 10^–10^; r_g †_ = 0.26, p_†_ = 1.01 × 10^–6^). Similarly, positive genetic correlations were observed between body fat percentage and overall lung cancer (r_g_ = 0.17, p = 6.11 × 10^–7^; r_g †_ = 0.17, p_†_ = 1.23 × 10^–6^) and squamous cell carcinomas (r_g_ = 0.23, p = 1.85 × 10^–7^; r_g †_ = 0.23, p_†_ = 9.81 × 10^–6^). Participant-reported activity level traits demonstrated negative genetic correlation with lung cancer susceptibility. Physical activity traits include DIY physical activity in last 4 weeks, exercise such as swimming or cycling in the last 4 weeks, as well as cycling or walking as methods of transport when going to work. Contrarily, having ‘no physical activity in the last 4 weeks’ demonstrated increased genetic correlation with lung cancer susceptibility. We highlight “swimming, cycling, and keeping fit in the last 4 weeks” which demonstrated significant negative genetic correlations with lung cancer susceptibility: overall lung cancer (r_g_ = − 0.33, p = 1.20 × 10^–9^; r_g †_ = − 0.33, p_†_ = 4.02 × 10^–10^), adenocarcinoma (r_g_ = − 0.26, p = 7.92 × 10^–6^; r_g †_ = − 0.25, p_†_ = 2.05 × 10^–5^), squamous cell carcinoma (r_g_ = − 0.32, p = 2.20 × 10^–7^; r_g †_ = − 0.33, p_†_ = 2.44 × 10^–6^), and ‘ever’ smokers (r_g_ = − 0.26, p = 3.46 × 10^–5^; r_g †_ = − 0.29, p_†_ = 1.72 × 10^–5^). A summary of the significant fitness-related associations is presented in Fig. 4.

### Heritability and genetic correlations between lung cancer and other specific traits

**Figure 5:**
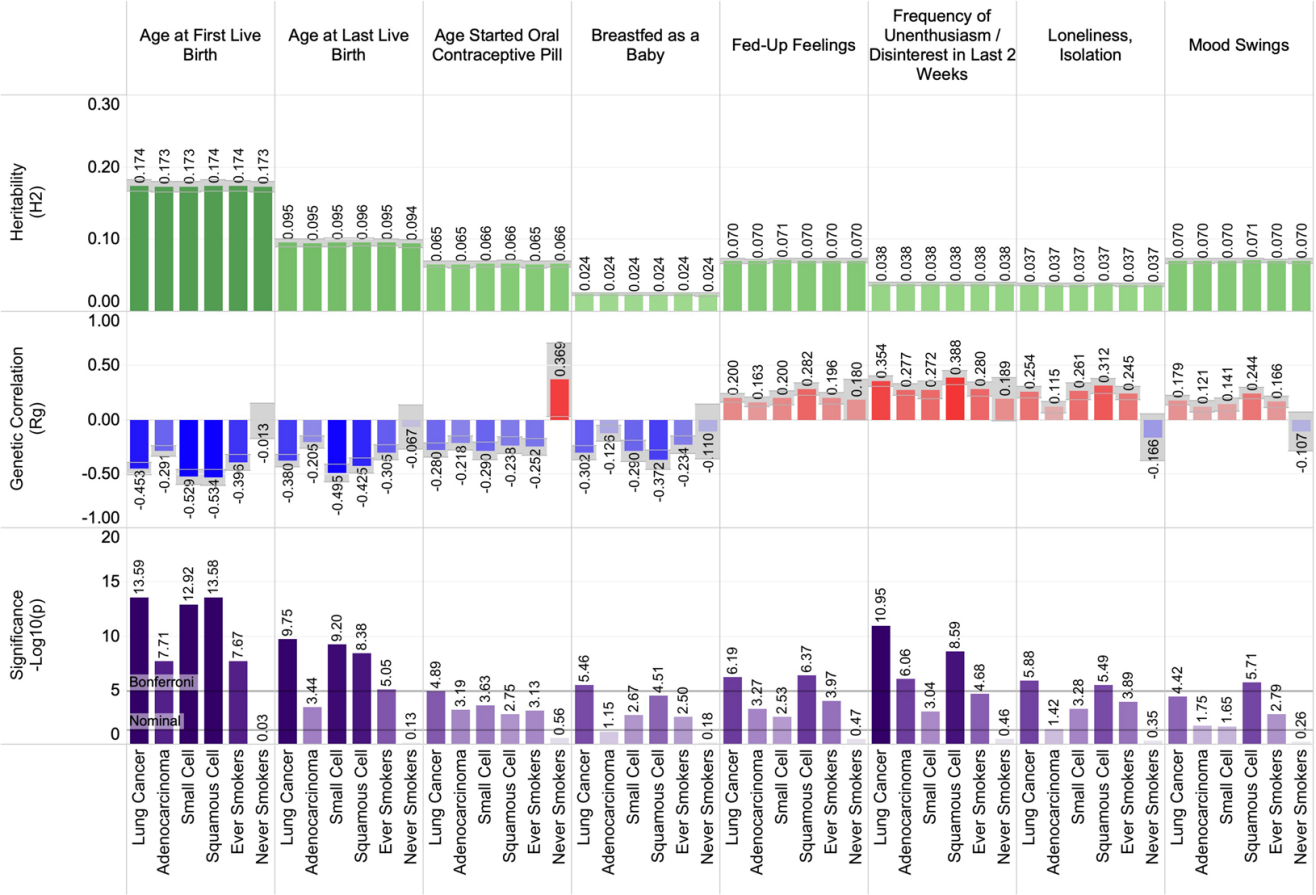
The shared heritability and genetic correlation between psychosocial and other specific traits with lung cancer.

**Figure 6:**
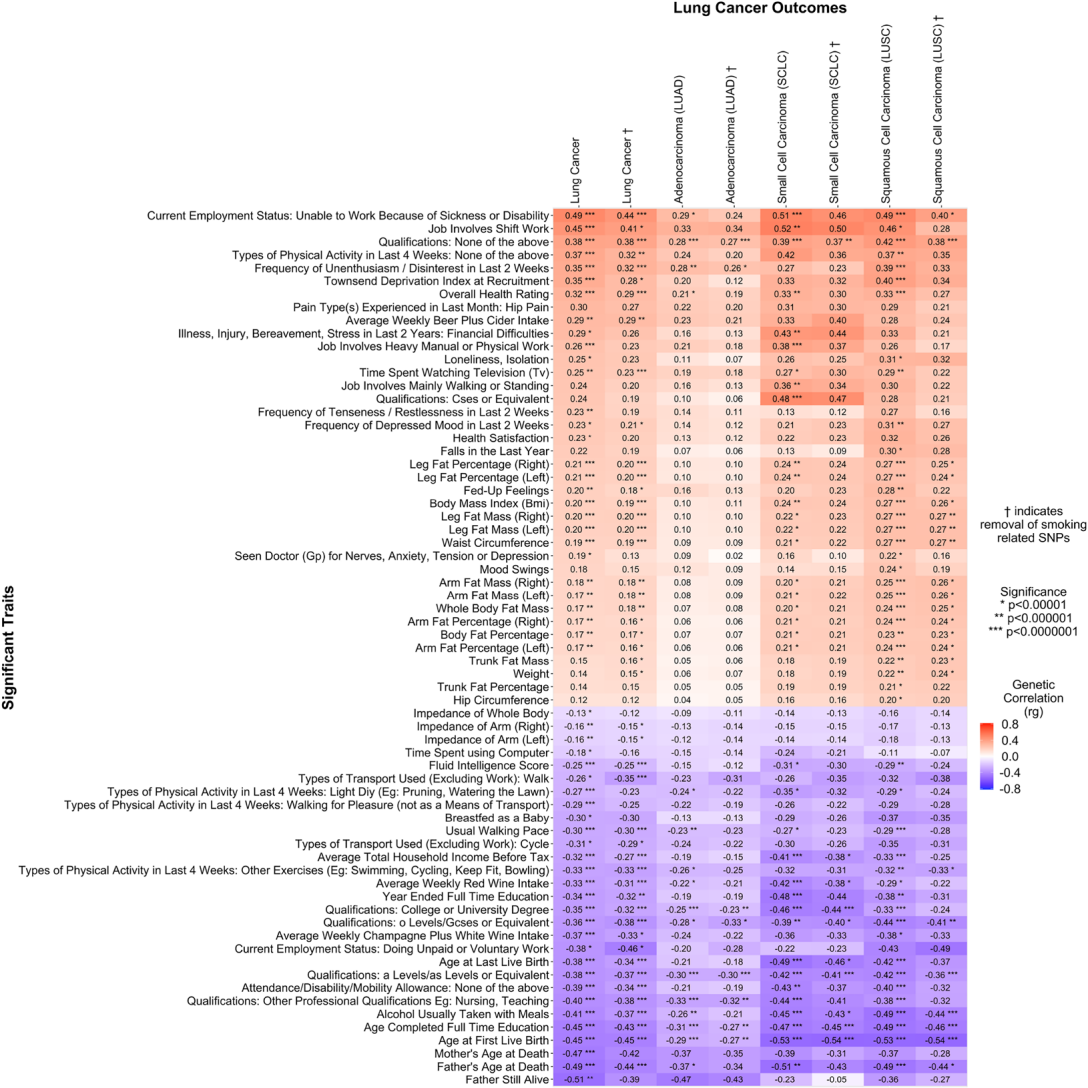
Genetic correlation plot of highly significant trait associations with lung cancer outcomes.

**Figure 7:**
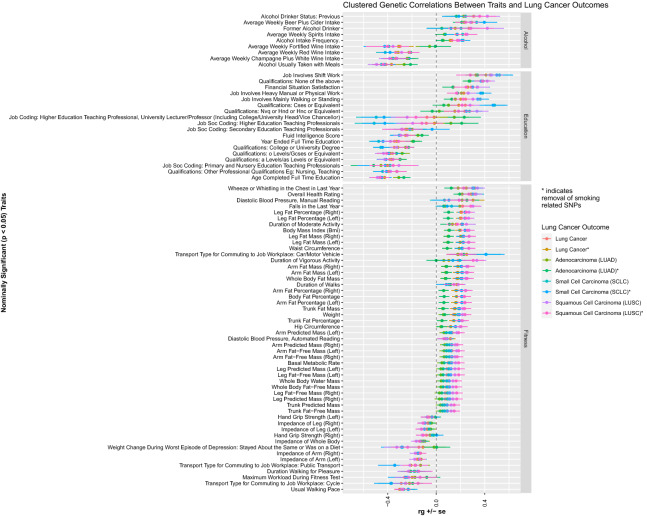
Overlapping forest plot of nominally significant trait associations with lung cancer outcomes clustered by alcohol use, educational ascertainment and fitness metrics.

**Figure 8:**
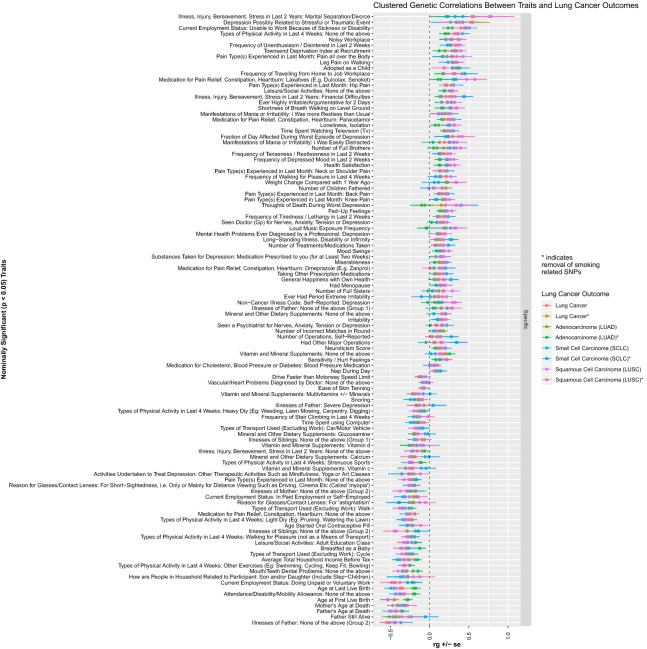
Overlapping forest plot of nominally significant trait associations with lung cancer outcomes clustered by specific traits.

Significant genetic correlation and heritability estimates were observed for select psychosocial traits. A participant’s reported ‘frequency of depressed mood in the last 2 weeks’ demonstrated a positive genetic correlation with lung cancer susceptibility for overall lung cancer (r_g_ = 0.23, p = 3.09 × 10^–6^; r_g †_ = 0.21, p_†_ = ﻿9.44 × 10^–6^). Specific depressive-related symptoms also demonstrated positive genetic correlation, including the frequency of uninthusiasm/disinterest in the last 2 weeks: overall lung cancer (r_g_ = ﻿0.35, p = 1.11 × 10^–11^; r_g †_ = 0.32, p_†_ = 1.40 × 10^–10^), adenocarcinoma (r_g_ = 0.28, p = 8.70 × 10^–7^; r_g †_ = ﻿0.26, p_†_ = 7.10 × 10^–6^), and squamous cell carcinoma (r_g_ = 0.39, p = ﻿2.54 × 10^–9^; r_g †_ = ﻿0.33, p_†_ = ﻿1.67 × 10^–5^). Being breastfed as a baby demonstrated a negative genetic correlation with lung cancer susceptibility. The genetic correlations for being breastfed as a baby were significant in the overall lung cancer (r_g_ = − 0.30, p = 3.46 × 10^–6^; r_g †_ = − 0.30, p_†_ = 3.50 × 10^–5^). In female only traits, both age at first live birth and age started oral contraceptive demonstrated negative genetic susceptivity with lung cancer. For age at first live birth the genetic predispositions for lung cancer are significant in overall lung cancer (r_g_ = ﻿﻿− 0.45, p = ﻿2.60 × 10^–14^; r_g †_ = 0.45, p_†_ = 4.98 × 10^–20^), adenocarcinoma (r_g_ = ﻿﻿− 0.29, p = ﻿﻿1.97 × 10^–8^; r_g †_ = ﻿﻿− 0.27, p_†_ = ﻿﻿1.78 × 10^–7^), small cell carcinoma (r_g_ = ﻿ ﻿− 0.53, p = ﻿1.20 × 10^–13^; r_g †_ = ﻿ ﻿− 0.54, p_†_ = ﻿1.78 × 10^–8^), squamous cell carcinoma (r_g_ = ﻿ ﻿− 0.53, p = ﻿ ﻿2.62 × 10^–14^; r_g †_ = ﻿ ﻿− 0.54, p_†_ = ﻿1.68 × 10^–11^), and ‘ever’ smokers (r_g_ = ﻿ ﻿− 0.40, p = 2.16 × 10^–8^; r_g †_ = − 0.43, p_†_ = 2.85 × 10^–11^). Similarly, the age of last live birth demonstrated also demonstrated a significant decrease in lung cancer susceptibility overall, and in the small cell, squamous cell and ever smoking cohorts. The trait ‘age started oral contraceptive’ bore significant genetic predispositions with overall lung cancer (r_g_ = − 0.28, p = ﻿1.30 × 10^–5^; r_g †_ = ﻿− 0.27, p_†_ = ﻿﻿ ﻿5.93 × 10^–5^). These findings are further detailed in Fig. 5. A full correlation plot of all highly correlated traits is presented as Fig. 6, which includes all UKBB traits with significant genetic correlation with lung cancer after a Bonferroni correction for statistical significance. Figures 7 and 8 presents all nominally associated UKBB traits (p < 0.05) including their r_g_ and standard errors in cohort clustered forest plots.

## Discussion

We sought to determine the shared genetic architecture between environmental and behavioral factors and lung cancer predisposition. LDSR has previously demonstrated efficacy and accuracy in determining the shared heritability and genetic correlation between phenotypes and disease states of interest^42,43^. To date, the TRICL-OncoArray Lung consortium comprises the largest lung cancer GWAS conducted in European-ancestry populations^30^. We leveraged these lung cancer GWAS meta-analysis data with GWAS summary statistics of traits from the UKBB to comprehensively assess shared genetic architectures between specific traits and lung cancer risk, observing numerous significant associations that were consistent across strata of lung cancer histology.

We observed significant positive and negative (*i.e*., protective) genetic correlations between lung cancer risk and individual behavioral characteristics and other environmental factors. We acknowledge that the strength of the LDSR method relies on the assumption that the genetic architectures between populations are similar. To ensure this, our analyses were conducted on European-ancestry populations in all studies, and SNPs included are those imputed using standard methods developed for application to the 1000 genomes project.

We provide further evidence that lung cancer is a heritable disease. Overall, our analysis estimated the heritability of lung cancer to be 8.3 ± 1.3%, with comparable heritability in adenocarcinoma (6.8 ± 1.0%), higher heritability in small cell lung carcinoma (10.5 ± 1.9%), and lower heritability in squamous cell carcinoma of the lung (5.2 ± 1.1%). These findings are similar to previous reports^29^. The heritability of lung cancer among never smokers was considerably lower than among smokers, which might indicate heterogeneity in etiology of lung cancer in never smokers obscures its heritable nature. It is noteworthy that we found no significant associations in LDSR analyses among the 'never' smoker's subgroup, but the observed genetic correlations in this cohort consistently mirroring the direction observed in 'ever' smokers and across histological subgroups. The never-smoker subgroup was a considerably smaller sample (2355 lung cancer cases, 7504 non-cancer controls) and had the lowest heritability of any of our lung cancer sub-strata, indicating that we may have been underpowered to detect cross-trait associations with this group.

The frequency and circumstance of alcohol consumption demonstrated a significant and mixed correlation with the genetic architecture of lung cancer. We found that "alcohol taken with meals" was negatively correlated with overall lung cancer. However, when analyzing this trend by type of alcohol consumed, higher average weekly beer and cider intake and higher weekly spirits intake were positively genetically correlated with lung cancer risk. In contrast, higher average weekly champagne, white wine, or red wine intake had a negative correlation. This effect has previously been observed through non-genetic epidemiological meta-analysis^44^, and, notably, we observe concordant findings through LDSR. One possible explanation is that concurrent smoking consumption is more likely in those who drink beer or partake in spirits and less likely in wine drinkers, possibly due to socioeconomic differences^45^. Evidence against this hypothesis includes that the genetic correlations with lung cancer and alcohol intake were consistent across histological subtypes and when contrasted against 'never' versus 'ever' smoking status, although non-significant in 'never' smokers.

Educational attainment traits demonstrated a consistent genetic correlation with lung cancer risk in LDSR analyses. Certifications of educational attainment were consistently negatively correlated with lung cancer susceptibility. The corollary is also true, with 'no educational qualifications' (i.e., no college or university degree), no professional qualifications in nursing or teaching, no "A" levels, and no general certificate of secondary education, demonstrating a positive correlation with lung cancer risk. These findings retained significance across histological subtypes. Removal of smoking-related SNPs as a method to mitigate residual confounding effects did not change the identified correlations or significance of these findings. Complementing these findings, it was independently found that fluid intelligence score, which had a consistent h^2^ ~ 0.22 ± 0.01, demonstrated a consistent negative genetic correlation with lung cancer across histological subtypes and smoking statuses.

Summary statistics for several quantitative as well as binary fitness-related traits demonstrated consistent associations. However, consistency in statistical significance was not achieved among each of the three histological subgroups. Indicators of BMI demonstrated relatively consistent findings. We highlight BMI and body fat percentage. These traits demonstrate significant heritability (h^2^ ~ 0.22 ± 0.01) and have consistent positive genetic correlations with lung cancer. A general trend of negative genetic correlation between increased physical activity and lung cancer risk was observed; however, these findings had marginal estimated heritability at around ~ 0.03. BMI's causal role in lung cancer oncogenesis was recently validated using Mendelian randomization^6^, however the strength of association measured in this prior study varied by lung cancer histology.

Several *specific* traits stood out from these analyses. A modest correlation was observed for depression and depression-related psychosocial traits, including 'frequency of fed-up feelings,' 'frequency of uninthusiasm/disinterest,' and 'loneliness, isolation,' and 'mood swings.' These captured symptoms are part of the diagnostic criteria for mental illnesses, and it is worth noting that the incidence of smoking behavior in populations who suffer from mental illness is higher than those without mental illnesses^46^. Other specific standout traits genetically correlated with lung cancer risk included the participant-reported status of being breastfed as a baby. The heritability of this trait was 0.023 ± 0.002, however, a consistent negative genetic correlation with lung cancer was observed. While interesting, these findings were only significant after correction for multiple comparisons testing for the overall and squamous cell lung cancer histological subgroups. The age at which a woman undergoes her 1st and last live birth and the age she started oral contraceptives were other specific traits that demonstrated a genetic correlation with lung cancer risk. These traits each revealed appreciable trait heritability and consistent, highly significant negative genetic correlations. It is well known that the ages of first live birth^47^, last live birth^48^, and initiation of oral contraceptive pills^49^ are associated with androgen modulation and modified cancer risk. It is logical that these traits are annotating such a reality in lung cancer predisposition^50,51^. We note that these results should be viewed as revealing only genetic associations, not for causal effect estimation.

Our use of LDSR, with an intercept, allowed for acceptable mitigation of population stratification and cryptic relatedness confounders that could exist between the UKBB population and our TRICL-OncoArray lung cancer dataset. Further, we used individuals of European descent in these cohorts to mitigate this risk. Additional confounding, predominantly through smoking, have the potential to limit the strength of these analyses. To appreciate any hidden effects of smoking, we sub-stratified our analysis by those who had and had not smoked roughly 100 cigarettes in their lifetime. In addition to this 'never' versus 'ever' smoking comparison, we re-ran LDSR analyses after excluding genomic regions previously associated with smoking-related behaviors. Although GWAS meta-analyses of smoking behaviors have included upwards of 500,000 individuals, it is likely that additional genetic loci of small effect influence smoking behaviors and remain undetected by GWAS. Therefore, our analyses excluding known smoking-associated regions may not fully account for the contribution of smoking-associated genomic variation to our traits in our LDSR analyses. We present all our results, including these smoking sub-analyses, in the supplemental material.

Using cross trait LDSR, we have identified positively and negatively correlated traits with lung cancer. These findings indicate that shared genetic backgrounds exist between these traits, including alcohol use, educational attainment, fitness, and several other specific traits with lung cancer development. Our work should be viewed as a considerable step towards understanding the shared genetic architecture between these traits and lung cancer. A potential next step in future investigations is to perform causal analyses on strongly correlated traits we have described. Mendelian randomization studies may help determine causal versus mere association between these traits and the development of lung cancer. Ultimately identifying causal relationships may help to understand the shared genetic architecture of these traits with lung cancer, as well as to accurately create predictive risk models for lung cancer development. While causal modeling has an important role, it requires identifying and specifying sets of markers that can reliably represent intermediate traits. The LD Score regression approach evaluates the entire genome and so should be a more powerful filter for future causal modeling, once adequate genetic predictors for each of the traits that have been identified in our analysis are available.

## Supplementary Information

Supplementary Information 1.

Supplementary Information 2.

## References

[1] Siegel RL, Miller KD, Jemal A (2019). Cancer statistics, 2019. CA Cancer J. Clin..

[2] Bosse Y, Amos CI (2018). A decade of GWAS results in lung cancer. Cancer Epidemiol. Biomarkers Prev..

[3] Bailey-Wilson JE, Amos CI, Pinney SM (2004). A major lung cancer susceptibility locus maps to chromosome 6q23-25. Am. J. Hum. Genet..

[4] Marant Micallef C, Shield KD, Baldi I (2018). Occupational exposures and cancer: A review of agents and relative risk estimates. Occup. Environ. Med..

[5] Rosell R, Karachaliou N (2016). Large-scale screening for somatic mutations in lung cancer. Lancet.

[6] Zhou W, Liu G, Hung RJ (2020). Causal relationships between body mass index, smoking and lung cancer: Univariable and multivariable Mendelian randomization. Int. J. Cancer..

[7] Pershagen G, Akerblom G, Axelson O (1994). Residential radon exposure and lung cancer in Sweden. N. Engl. J. Med..

[8] Hodge AM, Bassett JK, Shivappa N (2016). Dietary inflammatory index, Mediterranean diet score, and lung cancer: A prospective study. Cancer Causes Control..

[9] Doll R (1978). Atmospheric pollution and lung cancer. Environ. Health Perspect..

[10] Pershagen G (1985). Lung cancer mortality among men living near an arsenic-emitting smelter. Am. J. Epidemiol..

[11] Lissowska J, Bardin-Mikolajczak A, Fletcher T (2005). Lung cancer and indoor pollution from heating and cooking with solid fuels: The IARC international multicentre case-control study in Eastern/Central Europe and the United Kingdom. Am. J. Epidemiol..

[12] Dela Cruz CS, Tanoue LT, Matthay RA (2011). Lung cancer: Epidemiology, etiology, and prevention. Clin. Chest Med..

[13] Tokuhata GK, Lilienfeld AM (1963). Familial aggregation of lung cancer in humans. J. Natl. Cancer Inst..

[14] Tokuhata GK, Lilienfeld AM (1963). Familial aggregation of lung cancer among hospital patients. Public Health Rep..

[15] Sellers TA, Bailey-wilson JE, Elston RC (1990). Evidence for mendelian inheritance in the pathogenesis of lung cancer. J. Natl. Cancer Inst..

[16] Sellers TA, Chen PL, Potter JD, Bailey-Wilson JE, Rothschild H, Elston RC (1994). Segregation analysis of smoking-associated malignancies: Evidence for Mendelian inheritance. Am. J. Med. Genet..

[17] Sellers TA, Ooi WL, Elston RC, Chen VW, Bailey-wilson JE, Rothschild H (1987). Increased familial risk for non-lung cancer among relatives of lung cancer patients. Am. J. Epidemiol..

[18] Dragani TA, Manenti G, Pierotti MA (1996). Polygenic inheritance of predisposition to lung cancer. Ann. Ist. Super Sanita..

[19] Yang J, Weedon MN, Purcell S (2011). Genomic inflation factors under polygenic inheritance. Eur. J. Hum. Genet..

[20] Truong T, Hung RJ, Amos CI (2010). Replication of lung cancer susceptibility loci at chromosomes 15q25, 5p15, and 6p21: A pooled analysis from the international lung cancer consortium. J. Natl. Cancer Inst..

[21] Hung RJ, McKay JD, Gaborieau V (2008). A susceptibility locus for lung cancer maps to nicotinic acetylcholine receptor subunit genes on 15q25. Nature.

[22] Thorgeirsson TE, Geller F, Sulem P (2008). A variant associated with nicotine dependence, lung cancer and peripheral arterial disease. Nature.

[23] Timofeeva MN, Hung RJ, Rafnar T (2012). Influence of common genetic variation on lung cancer risk: Meta-analysis of 14 900 cases and 29 485 controls. Hum. Mol. Genet..

[24] Bulik-Sullivan B, Loh PR, Finucane HK (2015). LD score regression distinguishes confounding from polygenicity in genome-wide association studies. Nat. Genet..

[25] Maher B (2008). The case of the missing heritability. Nature.

[26] Tradigo G, Vacca R, Manini T, et al. A new approach to disentangle genetic and epigenetic components on disease comorbidities: Studying correlation between genotypic and phenotypic disease networks. In *Procedia Computer Science,* Vol. 110, 453–458 10.1016/j.procs.2017.06.119 (Elsevier B.V., 2017). 10.1016/j.procs.2017.06.119 PMC7172629 32318124

[27] Rubio-Perez C, Guney E, Aguilar D (2017). Genetic and functional characterization of disease associations explains comorbidity. Sci. Rep..

[28] Bulik-Sullivan B, Finucane HK, Anttila V (2015). An atlas of genetic correlations across human diseases and traits. Nat. Genet..

[29] McKay JD, Hung RJ, Han Y (2017). Large-scale association analysis identifies new lung cancer susceptibility loci and heterogeneity in genetic susceptibility across histological subtypes. Nat. Genet..

[30] Amos CI, Dennis J, Wang Z (2017). The OncoArray Consortium: A network for understanding the genetic architecture of common cancers. Cancer Epidemiol. Biomarkers Prev..

[31] Sudlow C, Gallacher J, Allen N (2015). UK Biobank: An open access resource for identifying the causes of a wide range of complex diseases of middle and old age. PLoS Med..

[32] Altshuler DM, Durbin RM, Abecasis GR (2012). An integrated map of genetic variation from 1,092 human genomes. Nature.

[33] Byun J, Han Y, Gorlov IP, Busam JA, Seldin MF, Amos CI (2017). Ancestry inference using principal component analysis and spatial analysis: A distance-based analysis to account for population substructure. BMC Genomics.

[34] Zhang LR, Morgenstern H, Greenland S (2015). Cannabis smoking and lung cancer risk: Pooled analysis in the International Lung Cancer Consortium. Int. J. Cancer..

[35] Schuller HM (2019). The neuro-psychological axis of smoking-associated cancer. J. Immunol. Sci..

[36] Hecht SS. Tobacco and cancer: Approaches using carcinogen biomarkers and chemoprevention. In *Annals of the New York Academy of Sciences*, Vol. 833, 91–111. 10.1111/j.1749-6632.1997.tb48596.x (Blackwell Publishing Inc., 1997). 10.1111/j.1749-6632.1997.tb48596.x 9616743

[37] Leon ME, Peruga A, McNeill A (2015). European code against cancer, 4th edition: Tobacco and cancer. Cancer Epidemiol..

[38] Amos CI, Pinney SM, Li Y (2010). A susceptibility locus on chromosome 6q greatly increases lung cancer risk among light and never smokers. Cancer Res..

[39] Liu M, Jiang Y, Wedow R (2019). Association studies of up to 1.2 million individuals yield new insights into the genetic etiology of tobacco and alcohol use. Nat. Genet..

[40] Jiang Y, Chen S, McGuire D (2018). Proper conditional analysis in the presence of missing data: Application to large scale meta-analysis of tobacco use phenotypes. PLOS Genet..

[41] Townsend P (1972). Deprivation. Health Visit..

[42] Fuller T, Reus V (2019). Shared genetics of psychiatric disorders [version 1; peer review: 2 approved]. F1000Research..

[43] Hartz SM, Horton AC, Hancock DB (2018). Genetic correlation between smoking behaviors and schizophrenia. Schizophr. Res..

[44] Chao C (2007). Associations between beer, wine, and liquor consumption and lung cancer risk: A meta-analysis. Cancer Epidemiol. Biomarkers Prev..

[45] Friedman GD, Tekawa I, Klatsky AL, Sidney S, Armstrong MA (1991). Alcohol drinking and cigarette smoking: An exploration of the association in middle-aged men and women. Drug Alcohol Depend..

[46] Lasser K, Boyd JW, Woolhandler S, Himmelstein DU, McCormick D, Bor DH (2000). Smoking and mental illness: A population-based prevalence study. J. Am. Med. Assoc..

[47] MacMahon B, Cole P, Lin TM (1970). Age at first birth and breast cancer risk. Bull World Health Organ..

[48] Setiawan VW, Pike MC, Karageorgi S (2012). Age at last birth in relation to risk of endometrial cancer: Pooled analysis in the epidemiology of endometrial cancer consortium. Am. J. Epidemiol..

[49] Marchbanks PA, McDonald JA, Wilson HG (2002). Oral contraceptives and the risk of breast cancer. N. Engl. J. Med..

[50] Siegfried JM, Hershberger PA, Stabile LP (2009). Estrogen receptor signaling in lung cancer. Semin. Oncol..

[51] Kawai H, Ishii A, Washiya K (2005). Estrogen receptor α and β are prognostic factors in non-small cell lung cancer. Clin. Cancer Res..

